# Catechol O-methyltransferase (*COMT*) functional haplotype is associated with recurrence of affective symptoms: A prospective birth cohort study

**DOI:** 10.1016/j.jad.2017.12.044

**Published:** 2018-03-15

**Authors:** Shinsuke Koike, Darya Gaysina, Peter B. Jones, Andrew Wong, Marcus Richards

**Affiliations:** a MRC Unit for Lifelong Health and Ageing at UCL, 33 Bedford Place, London WC1B 5JU, UK; b University of Tokyo Institute for Diversity & Adaptation of Human Mind (UTIDAHM), 3-8-1 Komaba, Meguro-ku, Tokyo 153-8902, Japan; c Center for Evolutionary Cognitive Sciences, Graduate School of Arts and Sciences, The University of Tokyo, 3-8-1 Komaba, Meguro-ku, Tokyo 153-8902, Japan; d International Research Center for Neurointelligence (WPI-IRCN), UTIAS, The University of Tokyo, 7-3-1 Hongo, Bunkyo-ku, Tokyo 113-8654, Japan; e EDGE Lab, School of Psychology, University of Sussex, Pevensey 1, Falmer, Brighton BN1 9QH, UK; f Department of Psychiatry, University of Cambridge, Cambridge CB2 0SZ, UK; g CAMEO, Cambridgeshire & Peterborough NHS Foundation Trust, Cambridge CB21 5EF, UK

**Keywords:** Life course epidemiology, Depression, Genetic association studies, Birth cohort

## Abstract

**Background:**

Catechol-*O*-methyltransferase (*COMT*) polymorphisms play an essential role in dopamine availability in the brain. However, there has been no study investigating whether a functional four-SNP (rs6269-rs4633-rs4818-rs4680) haplotype is associated with affective symptoms over the life course.

**Methods:**

We tested this using 2093 members of the Medical Research Council National Survey of Health and Development (MRC NSHD), who had been followed up since birth in 1946, and had data for *COMT* genotypes, adolescent emotional problems (age 13–15) and at least one measure of adult affective symptoms at ages 36, 43, 53, or 60–64 years. First, differences in the levels of affective symptoms by the functional haplotype using SNPs rs6269, rs4818, and rs4680 were tested in a structural equation model framework. Second, interactions between affective symptoms by *COMT* haplotype were tested under an additive model. Finally, a quadratic regressor (haplotype^2^) was used in a curvilinear model, to test for a possible inverted-U trend in affective symptoms according to *COMT*-related dopamine availability.

**Results:**

Women had a significant interaction between *COMT* haplotypes and adolescent emotional problem on affective symptoms at age 53. Post hoc analysis showed a significant positive association between adolescent emotional problems and affective symptoms at age 53 years in the middle dopamine availability group (valA/valB or met/met; *β* = .11, *p* = .007). For men, no significant interactions were observed.

**Conclusions:**

Combination of the *COMT* functional haplotype model and inverted-U model may shed light on the effect of dopaminergic regulation on the trajectory of affective symptoms over the life course.

## Highlights

• *COMT* polymorphisms play an essential role in dopamine availability in the brain.
• Association between *COMT* haplotype and depression over life course has not explored.
• We showed middle dopamine availability are more recurrence of depression.
• Future studies need to consider combination of *COMT* haplotype and inverted-U models.

## 1 Introduction

Affective symptoms are common psychological occurrences that mainly emerge in early adolescence (Patton et al., 2014, Thapar et al., 2012). Although most episodes remit and generally decrease with age (Patton et al., 2014), some people with adolescent affective symptoms will also experience affective symptoms in adulthood (Colman et al., 2014, Thapar et al., 2012). Genetic factors play an important role in the recurrent risk of affective symptoms, with specific genetic variants contributing to the onset and severity of these disorders via interactions with adolescent experiences (Antypa et al., 2013). Gene-environment, or more specifically, gene-behaviour interaction research has great potential to elucidate the aetiology of affective disorders (Rutter et al., 2006) and facilitate more personalised and, therefore, more effective intervention strategies to mitigate course of mental illness (Klengel and Binder, 2011).

Dopamine is a neurotransmitter that regulates a wide range of psychological function including motivation and reward activity (Antypa et al., 2013). Catechol-*O*-methyltransferase (*COMT*) is an enzyme in catecholamine metabolism that plays an essential role in brain dopamine availability. There are numerous genetic association studies implicating the *COMT* rs4680 (Val^158^Met) polymorphism in the incidence of major depression and anxiety disorders, and response to antidepressants (Antypa et al., 2013, Gatt et al., 2015). A population based study found that Met-allele carriers with childhood family-related adversities were more likely to have clinical depressive symptoms (Aberg et al., 2011). On the other hand, in a prospective longitudinal study neuroticism (a personality trait that highly correlates with depression) significantly increased from adolescence to adulthood in female Val homozygotes, but not in Met-allele carriers (Lehto et al., 2013). These results suggest that life course trajectories of affective symptoms can be modified by the *COMT* polymorphisms; however, results are inconsistent.

A recent protein structure study showed that *COMT* activity is converted into three enzyme levels by the four-SNP (rs6269-rs4633-rs4818-rs4680) (Nackley et al., 2006). This functional haplotype is associated with pain sensitivity, probably because *COMT* regulates catecholamine and encephalin levels in the brain (Aggarwal et al., 2011, Diatchenko et al., 2005).

Studies have shown that middle dopamine availability by the COMT haplotype (valA/valB or met/met) was associated with the highest verbal IQ performance at age 8 years (Barnett et al., 2009) but not with cognitive performance at age 15 years (Gaysina et al., 2013). Another study found that adolescents and young adults with middle dopamine availability (valA/met) had the largest white matter volume in the prefrontal cortex (Liu et al., 2010). This suggests that cognitive function may be associated with an optimal dopamine density in the prefrontal cortex, according to an inverted-U shape model (Takahashi, 2013). It therefore may be possible that this pattern is also observed for affective symptoms; however, to the best of our knowledge this has not been investigated. However, to the best of our knowledge this has not been investigated, and evidence is lacking on how life course trajectories of affective symptoms are modified by *COMT*.

The MRC National Survey of Health and Development (NSHD) is the oldest prospective birth cohort study in the world, beginning in 1946 in England, Scotland, and Wales. This cohort has measured symptoms of affective symptoms from adolescence through middle age (Colman et al., 2007). In the present study, we tested whether the *COMT* four-SNP functional haplotype was associated with affective symptoms from adolescence to old age under an additive and curvilinear model.

## 2 Methods

### 2.1 Participants

**Fig. S1:**
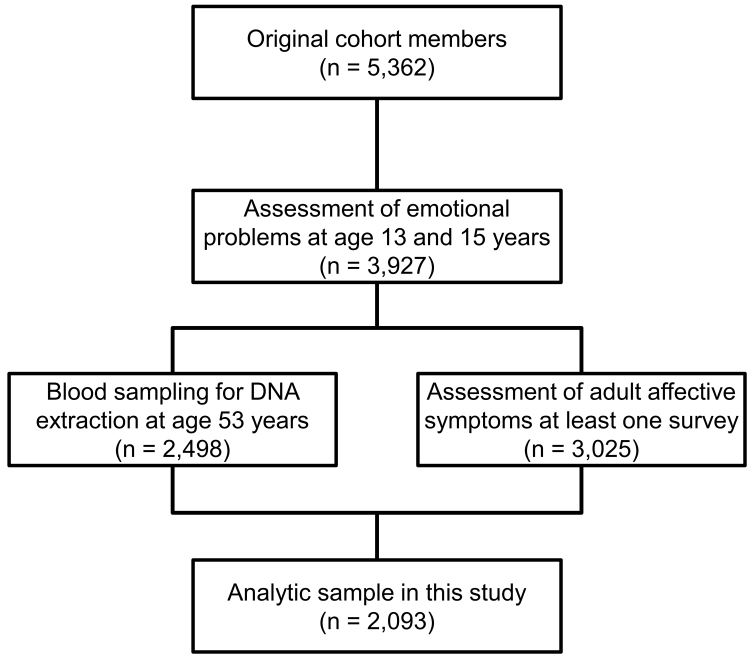
Participants flow in this study.

The NSHD initially consisted of a socially-stratified sample of 5362 children from all single births within marriage during 1 week in March 1946 in England, Scotland, and Wales. Reflecting the demographic characteristic of the British population in 1946, all of study members are of White British ethnicity. At ages 13 and 15 years, 3927 study members were rated for emotional problems by their teachers (Supplementary Fig. S1). At age 36, 43, 53, and 60–64 years, 3293, 3157, 2902, and 2185 study members, respectively, responded to questions eliciting affective symptoms (see Measures). Blood samples for DNA extraction were collected from 2756 study members at age 53 years, and genotyping were conducted on 2498 of these samples. From these denominators the analytic sample consisted of 2093 study members with non-missing data for *COMT* genotyping, adolescent emotional problems, and at least one measure of adult affective symptoms. Those included in the analyses were more likely to be female (*p* = .003) and had fewer adolescent emotional problems (*p* < .004) compared to those excluded. However, there were no differences at the 5% level in affective symptoms at ages 36, 53 and 60–64 years. At ages 53 and 60–64 the cohort was representative in most respects to the national population of a similar age, as compared to Census (Stafford et al., 2013, Wadsworth et al., 2003) and Integrated Household Survey data (Stafford et al., 2013).

Ethical approval for this study was obtained from the Greater Manchester Central and Scottish A Research Ethics committees. All study members gave written informed consent.

### 2.2 Measures

#### 2.2.1 Affective symptoms

Behaviour, mood and emotions in adolescence (at ages 13 and 15) were rated by teachers using forerunners of the Rutter A scale (Elander and Rutter, 1996, Rutter et al., 1970). These ratings have been classified into 3 behavioral dimensions reflecting emotional (internalizing) problems (e.g. extremely fearful); conduct (externalizing) problems (e.g. a quarrelsome and aggressive child); and self-control (e.g. a poor worker or lazy) (Koike et al., 2016, Nishida et al., 2014, Xu et al., 2013). We used the emotional problem score to represent adolescent emotional problems, which was calculated from the standardized mean of the factor scores at both ages, with appropriate goodness of fit statistics indicating satisfactory fit (Xu et al., 2013). In adulthood affective symptoms were assessed at ages 36, 43, 53 and 60–64. Affective symptoms were assessed at age 36 years using a short version of the Present State Examination (PSE), a clinically validated interview administrated by trained nurses (range 1―7) (Wing et al., 1974). The index of association (which correlates highly with kappa) between nurse and expert ratings at the syndrome level based on 526 recorded NSHD interviews averaged 0.71; and agreement was 0.74 for Index of Definition assignment (Rodgers and Mann, 1986). The index of definition score was intended to detect nervous or emotional symptoms and had statistically one factor (Rodgers and Mann, 1986). At age 43 years, the Psychiatric Symptom Frequency (PSF) scale was administered by interview using an 18-item questionnaire (range 0―90, Cronbach's α = .88) (Lindelow et al., 1997). For these assessments, we used the original scores. At ages 53 and 60–64 years, the 28-item version of the self-report General Health Questionnaire (GHQ-28) was used (Goldberg and Hillier, 1979). Each item was scored using a 4-level Likert scale recoded to 0-0-1-1 and re-summed (range 0―28, α = .92 and .89, respectively). The GHQ-28 had statistically four factors as well as one factor which was correlated with other affective symptom measures such as the Beck Depression Inventory and the Center for Epidemiological Studies Depression scales (Sakakibara et al., 2009).

#### 2.2.2 Genotyping

DNA was extracted and purified from whole blood using the Puregene DNA Isolation Kit (Flowgen, Leicestershire, UK) according to the manufacturer's protocol (Rousseau et al., 2006). Since a SNP rs4633 does not change the amino acid sequence (CAC [histidine] to CAT [histidine]) well-known as a benign allele and contributes to no alteration of dopamine availability, SNPs rs6269, rs4818, and rs4680 were typed by using the KASPar system by KBioscience, UK (http://www.kbioscience.co.uk). Integrity of the genotyping was checked by genotypeing frequency, concordance between duplicate samples (> 95% concordance) and Hardy-Weinberg equilibrium (*p* > 0.05).

### 2.3 Statistical analysis

**Fig. 1:**
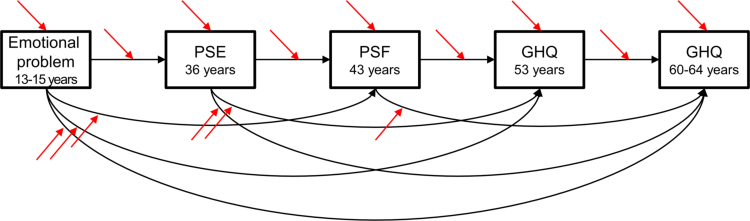
**Initial structural equation model (SEM) fitted to the trajectory of affective symptoms.** Abbreviations: PSE, a short version of the Present State Examination; PSF, the Psychiatric Symptom Frequency scale; GHQ-28, the 28-item version of the General Health Questionnaire. An SEM was set to test the trajectory of affective symptoms shown by black arrows and the main effects of a modifier and the interactions of affective symptoms by a modifier shown by red arrows. First, we used sex as a modifier to explore sex difference in the trajectory of affective symptoms. Second, we used the *COMT* functional haplotype under an additive curvilinear model using a quadratic regressor (haplotype^2^), to test for a possible inverted-U trend in affective symptoms according to dopamine availability. (For interpretation of the references to color in this figure legend, the reader is referred to the web version of this article.)

Three SNPs were in linkage disequilibrium (rs6269―rs4680 and rs4818―rs4680, *r*^*2*^ = 0.72; rs6269―rs4818, *r*^*2*^ = 0.97), and were set into three functional haplotypes (valA, G-G-G; met, A-C-A; valB, A-C-G) as suggested in the original study (Nackley et al., 2006). Since the met allele has middle dopamine availability in-between the valA and valB allele levels (Nackley et al., 2006), study members were then categorized into five diplotype groups: the highest dopamine availability (valA/valA), second highest (valA/met), middle (valA/valB or met/met), second lowest (valB/met), and lowest (valB/valB) (Barnett et al., 2009, Gaysina et al., 2013). We tested for the presence of an inverted-U trend in affective symptoms according to the *COMT* haplotype. First, a saturated structural equation model (SEM) was fitted for the trajectory of affective symptoms, including the sex interactions (Fig. 1) as previous studies have shown sex-specific effects (Hatch et al., 2007). Interactions were calculated using the numerical variables (sex: male=1, female=2; haplotype: the highest=1, the lowest=5) and the “indProd” function in R. The estimation of the model was conducted using robust maximum likelihood estimation, and missing values were handled using full information maximum likelihood (FIML). Model fit indicated adequate fit (CFI) > 0.90, Tucker-Lewis index (TLI) > 0.90, root mean square error of approximation (RMSEA) < 0.10, and standardized root mean square residual (SRMR) < 0.08. SEM was performed using the “lavaan” package version 0.5–18 in R version 3.2.1 (R Core Team, 2014; Rosseel, 2012). Where sex interactions were significant in the model, further analyses were stratified by sex.

Next, we fitted the SEM model to test the trajectory of affective symptoms by the *COMT* functional haplotype under an additive curvilinear model using a quadratic regressor (haplotype^2^), to test for a possible inverted-U trend in symptoms according to dopamine availability (Fig. 1). Significant interactions between all associations for adolescent emotional problems and adult affective symptoms by the *COMT* haplotype were further tested using multiple-group analysis for the initial SEM model to estimate the correlations within each genotype group, using equal loadings, means, residuals, and intercepts for the non-significant effect of genetic modification.

## 3 Results

Descriptive statistics showed that female study members had higher scores than males on all affective measures, as expected (Table 1). There were no significant gender differences in the distribution of the *COMT* functional haplotypes. There was no significant difference in any measure of affective symptoms between functional haplotype (Table 2 and Supplementary Tables S1 and S2).

**Table 1:** Demographic characteristics and genotypes in this study.

	Total (n = 2093)	Male (n = 1045)	Female (n = 1048)	Gender differences P value^3^)
Affective symptoms, mean [SD]				
Adolescents emotional problem^1^)	−0.06 [0.97]	**−0.15 [0.95]**	**0.02 [0.98]**	**< .001**
PSE index of definition score at age 36 years	1.9 [1.2]	**1.7 [1.1]**	**2.1 [1.3]**	**< .001**
PSF total score at age 43 years	10.4 [10.1]	**8.7 [8.7]**	**12.1 [11.1]**	**< .001**
GHQ-28 score at age 53 years	2.5 [4.4]	**1.9 [3.8]**	**3.1 [4.9]**	**< .001**
GHQ-28 score at age 60–64 years	2.3 [3.7]	**1.7 [3.0]**	**2.8 [4.3]**	**< .001**
*COMT* functional haplotype^2^), n (%)				
Highest (valA/valA)	355 (17.1)	183 (17.7)	172 (16.5)	.94
Second highest (valA/met)	861 (41.5)	432 (41.7)	429 (41.3)	
Middle (valA/valB or met/met)	664 (32.0)	326 (31.5)	338 (32.5)	
Second lowest (valB/met)	182 (8.8)	88 (8.5)	94 (9.0)	
Lowest (valB/valB)	14 (0.7)	7 (0.7)	7 (0.7)	

Abbreviations: PSE, the Present State Examination; PSF, the Psychiatric Symptom Frequency; GHQ-28, the 28 item version of the General Health Questionnaire.

1) Z scores.

2) The functional haplotype was defined by three SNPs (rs6269, rs4818, and rs4680), and each participant was categorized into five in accordance with dopamine availability.

3) Gender differences were tested using t-test. Bold shows 5% level significance.

**Table 2:** Presence of affective symptoms and genotypes.

	Highest (valA/valA) (n = 355)	Second highest (valA/met) (n = 861)	Middle (valA/valB or met/met) (n = 664)	Second lowest (valB/met) (n = 182)	Lowest (valB/valB) (n = 14)	Group differences P value^1^)
Affective symptoms, mean [SD]						
Adolescents emotional problem	−0.04 (0.97)	−0.05 (0.98)	−0.09 (1.00)	−0.09 (0.87)	−0.28 (0.74)	.83
PSE index of definition score at age 36 years	2.0 (1.2)	1.9 (1.2)	1.9 (1.2)	1.9 (1.2)	1.9 (1.3)	.62
PSF total score at age 43 years	10.9 (9.2)	10.3 (10.6)	10.3 (9.8)	10.1 (10)	11.2 (8.3)	.86
GHQ-28 score at age 53 years	2.6 (4.4)	2.4 (4.4)	2.6 (4.4)	2.5 (4.2)	1.7 (3.0)	.78
GHQ-28 score at age 60–64 years	2.4 (3.8)	2.2 (3.7)	2.2 (3.7)	2.3 (3.7)	2.5 (3.2)	.95

The results for men and women are shown in supplementary Tables S1 and S2, respectively.

1) Group differences were tested using ANOVA.

The initial SEM model to test the effect of sex did not have a good fit (*n* = 2093, *χ*^*2*^ = 98.0, *p* < .001, CFI = .86, TLI = 0.52, RMSEA = .065, SRMR = 0.057) and showed a main effect of sex in all affective symptom measures, as well as sex interactions with the adolescent emotional problems on the GHQ score at age 60–64, and with the PSF score at age 43 on the GHQ score at age 60–64.

**Fig. 2:**
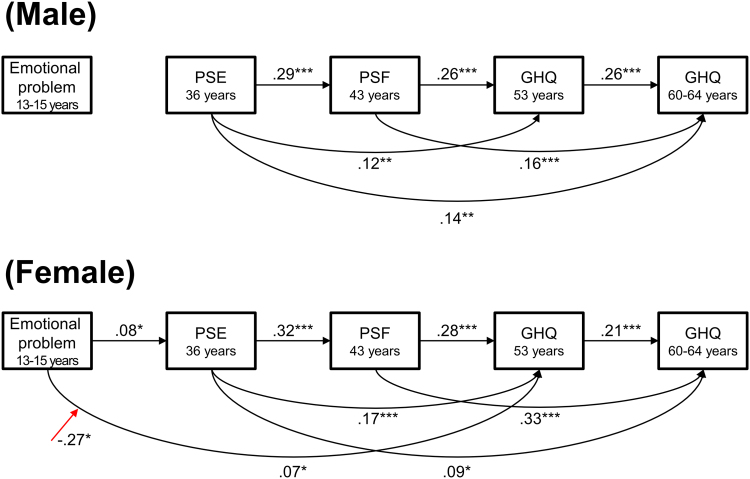
**The interaction of affective symptoms by the*****COMT*****haplotype.** Abbreviations: PSE, a short version of the Present State Examination; PSF, the Psychiatric Symptom Frequency scale; GHQ-28, the 28-item version of the General Health Questionnaire. Only significant relationships and interactions are shown with standardized coefficients (*p < .05, **p < .01, ***p < .001). The trajectory of affective symptoms and the interactions between adolescent emotional problems and the *COMT* functional haplotype (red arrow) are shown for men and women. The interaction between adolescent emotional problems and adult affective symptoms at age 53 years by the haplotype in women was indicated under an additive curvilinear model (Haplotype: β = .25, p = .036; haplotype^2^: β = −.27, p = .026). (For interpretation of the references to color in this figure legend, the reader is referred to the web version of this article.)

**Fig. 3:**
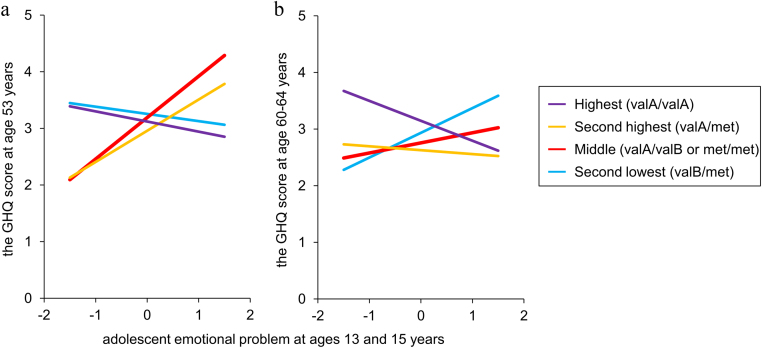
The relationship between adolescent emotional problem and the GHQ score at age 53 (a) and 60–64 (b) years in women for each haplotype. An intercept and a slope for the relationship in each haplotype group was estimated using multiple-group analysis for the initial SEM model with equal loadings, means, residuals, and intercepts for the non-significant effect of genetic modification.

The SEM models to test the COMT haplotype effect had a good fit for men (n = 1045, χ^2^ = 12.6, p = .90, TLI = 1.18, CFI = 1.0, RMSEA < .001, SRMR = 0.019) and women (n = 1048, χ2 = 21.7, p = .36, CFI = .99, TLI = 0.98, RMSEA = .009, SRMR = 0.016). The model for men showed no main effect of the *COMT* haplotype or significant interaction by the haplotype (*p* > .05). The model for women showed no main effect of the haplotype but showed a significant interaction between adolescent emotional problems and the haplotype (*β* = .25, *p* = .036) and haplotype^2^ (*β* = −.27, *p* = .026) on GHQ score at age 53 years (Fig. 2). Post hoc analysis showed a significant positive association between adolescent emotional problems and GHQ score at age 53 years only in the middle dopamine availability group (valA/valB or met/met; *β* = .11, *p* = .007; Fig. 3a), while there were no significant associations in the other haplotype groups (highest [valA/valA]: *β* = −.01, *p* = .95; second highest [valA/met]: *β* = .09, *p* = .080; second lowest [valB/met]: *β* = −.05, *p* = .44). We excluded females with the lowest dopamine availability group (valB/valB) from this analysis because this group was very small (*n* = 7). A significant interaction was not observed between adolescent emotional problems and the haplotype on GHQ score at age 60–64 years (Fig. 3b).

## 4 Discussion

A population representative prospective birth cohort study revealed that the *COMT* functional haplotype modifies the association between adolescent emotional problems and adult affective symptoms. These results suggest that females with a middle level of dopamine availability and high adolescent emotional problems are more likely to have recurrent affective symptoms in midlife. To the best of our knowledge, this is the first study to show that the *COMT* functional haplotype can alter affective symptom trajectory over approximately 50 years from adolescence to late midlife. These results suggest that a combination of the functional haplotype and inverted-U shape models can reveal genetic influence on the trajectory of affective symptoms through the life course.

The present study showed that females with middle dopamine availability (valA/valB or met/met) and evidence of emotional problems in adolescence were more likely to have affective symptoms at age 53. There are several possible reasons why the association occurred only in middle-aged females. Previous studies have suggested that those with middle dopamine ability have optimal childhood cognition and larger volume in the prefrontal cortex (Barnett et al., 2009, Liu et al., 2010). In NSHD, childhood cognition was inversely associated with the GHQ-28 at age 53 score after adjusting for educational attainment, early socioeconomic status (SES) and adverse circumstances, and adult SES, adverse circumstances and negative health behaviors (Hatch et al., 2007), but only in women. Second, the menopausal syndrome, triggered by reduced estrogen, may be associated with the *COMT* haplotype, since dopamine also influences sex hormone metabolism in the hypothalamic-pituitary-adrenal (HPA) axis, leading to reduced level of estrogen (Gordon et al., 2015). However, the relationship between estrogen level modified by *COMT* polymorphisms and psychological symptoms is complex, and requires further investigation. One reason why the modification disappeared again at age 60–64 years is that the decrease of sample size from age 53 years may have reduced the power and increase bias. Another reason is that after the critical period above explained, the increase of physical diseases and environmental factors such as retirement and bereavement may affect the psychological problems greater.

In the present study, two biological-based models, a curvilinear model stratified by the functional haplotype, may elucidate the association between *COMT* genotypes and affective symptoms. In particular, the association for the highest dopamine availability (valA/valA) group was in the opposite direction to that in the middle (valA/valB) group, both of which were typed as val/val on rs4680 genotype. A similar pattern was seen in the second highest (valA/met) and the second lowest (valB/met) groups. There have been numerous studies conducted using the *COMT* val^158^met polymorphism in psychiatry; however, results are inconsistent. Future gene-environment interaction researches will be conducted by using combinations of biological findings not merely gene type.

Several limitations should be considered. First, the disproportionate loss to follow-up such as greater adolescent emotional problem, which is typically associated with lower social advantage and poorer health, could have potentially affected the results. Second, although the study remained broadly representative of the mainland British population, the study sample is of White British ethnicity. While this reflects the demographic characteristic of the British population in 1946, the findings of this study cannot necessarily be extrapolated to other ethnic groups. Third, we did not use other *COMT* SNPs such as the rs737865 polymorphism, which is associated with cognitive function (Gaysina et al., 2013) and may affect the present findings. Forth, the different symptom scales over time could have affected the results. Fifth, power may still be limited for a genetic study although we used a relatively large sample, where power analysis showed that 0.079 of the slope could be detected in linear bivariate regression (*n* = 2093, *α* = .05, *β* = .95, two-tailed).

In conclusion, a population representative prospective birth cohort study revealed that females with middle dopamine availability by *COMT* functional haplotype and high adolescent emotional problem were more likely to have affective symptoms at age 53 years. These results indicate that the combination of the *COMT* functional haplotype and inverted-U models may shed further light on dopamine regulation of affective symptoms over the life course.
