# Supplementary material for: Catechol O-methyltransferase (COMT) functional haplotype is associated with recurrence of affective symptoms: A prospective birth cohort study
Source: J Affect Disord. 2018 Mar 15;229:437–42. doi: 10.1016/j.jad.2017.12.044 (PMC5814675; doi:10.1016/j.jad.2017.12.044)
Supplement: Supplementary file 1 — Supplementary material [file mmc1.docx]

Supplementary table S1. Presence of affective symptoms and genotypes in men.

|  | Highest  (valA/valA)  (n = 183) | Second highest  (valA/met)  (n = 432) | Middle  (valA/valB or met/met)  (n = 326) | Second lowest  (valB/met)  (n = 88) | Lowest  (valB/valB)  (n = 7) | Group differences  P value^1)^ |
| --- | --- | --- | --- | --- | --- | --- |
| Affective symptoms, mean [SD] |  |  |  |  |  |  |
| Adolescents emotional problem | -0.10 (0.95) | -0.14 (0.95) | -0.19 (0.98) | -0.14 (0.91) | -0.45 (0.60) | .77 |
| PSE index of definition score at age 36 years | 1.8 (1.1) | 1.7 (1.1) | 1.7 (1.0) | 1.6 (0.9) | 1.8 (1.6) | .58 |
| PSF total score at age 43 years | 9.4 (8.0) | 8.4 (8.8) | 8.8 (8.8) | 8.6 (9.2) | 10.1 (8.8) | .79 |
| GHQ-28 score at age 53 years | 2.2 (4.2) | 1.7 (3.8) | 1.9 (3.5) | 1.8 (3.7) | 2.9 (4.0) | .67 |
| GHQ-28 score at age 60-64 years | 1.6 (2.3) | 1.7 (3.3) | 1.6 (2.8) | 1.7 (3.3) | 3.3 (3.9) | .71 |

1) Group differences were tested using ANOVA.

Supplementary table S2. Presence of affective symptoms and genotypes in women.

|  | Highest  (valA/valA)  (n = 172) | Second highest  (valA/met)  (n = 429) | Middle  (valA/valB or met/met)  (n = 338) | Second lowest  (valB/met)  (n = 94) | Lowest  (valB/valB)  (n = 7) | Group differences  P value^1)^ |
| --- | --- | --- | --- | --- | --- | --- |
| Affective symptoms, mean [SD] |  |  |  |  |  |  |
| Adolescents emotional problem | 0.01 (0.98) | 0.04 (1.00) | 0.01 (1.01) | -0.04 (0.83) | -0.12 (0.87) | .96 |
| PSE index of definition score at age 36 years | 2.2 (1.3) | 2.2 (1.3) | 2.0 (1.3) | 2.2 (1.4) | 2.0 (1.2) | .60 |
| PSF total score at age 43 years | 12.4 (10.1) | 12.2 (11.9) | 11.7 (10.5) | 11.5 (10.6) | 12.3 (8.3) | .95 |
| GHQ-28 score at age 53 years | 3.1 (4.6) | 3.0 (4.9) | 3.2 (5.1) | 3.3 (4.6) | 0.6 (0.8) | .67 |
| GHQ-28 score at age 60-64 years | 3.2 (4.7) | 2.6 (4.0) | 2.8 (4.4) | 2.9 (4.1) | 1.3 (1.5) | .72 |

1) Group differences were tested using ANOVA.
